# Supplementary material for: Chagas cardiomyopathy in Boston, Massachusetts: Identifying disease and improving management after community and hospital-based screening
Source: PLoS Negl Trop Dis. 2024 Jan 19;18(1):e0011913. doi: 10.1371/journal.pntd.0011913 (PMC10830043; doi:10.1371/journal.pntd.0011913)
Supplement: S1 Table — Arrhythmias and conduction disease can occur from B1 through D stages. HF, heart failure; NYHA, New York Heart Association. Adapted from Nunes et al [2]. (DOCX) [file pntd.0011913.s001.docx]

**Supplemental Table 1. American Heart Association Classification of Chagas Cardiomyopathy**

| **Definitions and Progression of Chagas Disease** | | | | |  |
| --- | --- | --- | --- | --- | --- |
| ***Indeterminate Form*** | ***Chagas Cardiomyopathy*** | | | |  |
| A | B1 | Chagas Dilated Cardiomyopathy/Heart Failure | | |  |
| Patients at risk for developing heart failure. They have positive serology, neither structural cardiopathy nor HF symptoms. Normal ECG. | Patients with structural cardiopathy, evidenced by ECG or TTE changes, but with normal global ventricular function and neither current nor previous signs or symptoms of HF. | B2 | C | D |  |
|  |  | Patients with structural cardiopathy characterized by global ventricular dysfunction and neither current nor previous signs and symptoms of HF. | Patients with ventricular dysfunction and current or previous symptoms of HF  (NYHA functional class I, II, III, or IV). | Patients with refractory symptoms of HF at rest despite optimized clinical treatment requiring special interventions |  |
|  |  |  |  |  |  |
|  |  |  |  |  |  |
|  |  |  |  |  |  |
|  |  |  |  |  |  |
|  |  |  |  |  |  |
|  |  |  |  |  |  |
|  |  |  |  |  |  |
|  |  |  |  |  |  |
|  |  |  |  |  |  |
|  |  |  |  |  |  |
|  |  |  |  |  |  |
|  |  |  |  |  |  |
