# Supplementary material for: Chagas cardiomyopathy in Boston, Massachusetts: Identifying disease and improving management after community and hospital-based screening
Source: PLoS Negl Trop Dis. 2024 Jan 19;18(1):e0011913. doi: 10.1371/journal.pntd.0011913 (PMC10830043; doi:10.1371/journal.pntd.0011913)
Supplement: S2 Table — *NYHA, New York Heart Association. Adapted from Keegan et al [36]. (DOCX) [file pntd.0011913.s002.docx]

**Supplemental Table 2. Rassi Risk Score to Predict Mortality Related to Chagas Disease**

| **Risk Factor** | **Points** |
| --- | --- |
| NYHA* Class III or IV | 5 |
| Cardiomegaly (chest X-ray) | 5 |
| Segmental or global wall motion abnormalities (echocardiogram) | 3 |
| Non-sustained ventricular tachycardia (ambulatory monitor) | 3 |
| Low QRS voltage (electrocardiogram) | 2 |
| Male sex | 2 |

| Total Points | Total Mortality | | Risk |
| --- | --- | --- | --- |
|  | 5 years | 10 years |  |
| 0-6 | 2% | 10% | Low |
| 7-11 | 18% | 44% | Intermediate |
| 12-20 | 63% | 84% | High |
