# Supplementary material for: Chagas cardiomyopathy in Boston, Massachusetts: Identifying disease and improving management after community and hospital-based screening
Source: PLoS Negl Trop Dis. 2024 Jan 19;18(1):e0011913. doi: 10.1371/journal.pntd.0011913 (PMC10830043; doi:10.1371/journal.pntd.0011913)
Supplement: S3 Table — (DOCX) [file pntd.0011913.s003.docx]

**Supplemental Table 3: Proportion of ECG Abnormalities by Age Group**

| **Age Group** | **Number of Patients with Abnormal ECG – no. (%)** |
| --- | --- |
| >50 years old | 20/29 (70) |
| >40 and ≤ 50 | 18/29 (62) |
| >30 and ≤ 40 | 8/26 (31) |
| ≤ 30 | 4/18 (22) |
