# Supplementary material for: Chagas cardiomyopathy in Boston, Massachusetts: Identifying disease and improving management after community and hospital-based screening
Source: PLoS Negl Trop Dis. 2024 Jan 19;18(1):e0011913. doi: 10.1371/journal.pntd.0011913 (PMC10830043; doi:10.1371/journal.pntd.0011913)
Supplement: S4 Table — (DOCX) [file pntd.0011913.s004.docx]

**Supplemental Table 4: American Heart Association Stage of Cardiomyopathy by Age Group**

| **Age Group** | **Number of Patients in Each AHA Stage – no. (%)** | | | | |
| --- | --- | --- | --- | --- | --- |
|  | Stage A | Stage B1 | Stage B2 | Stage C | Stage D |
| >50 | 6/48 (13) | 15/30 (50) | 1/7 (14) | 3/8 (38) | 1 (100) |
| ≤ 50 | 42/48 (88) | 15/30 (50) | 4/7 (57) | 3/8 (38) | 0 |
| ≤ 40 | 31/48 (65) | 5/30 (17) | 2/7 (29) | 1/8 (13) | 0 |
| ≤ 30 | 13/48 (27) | 1/30 (3) | 0 | 1/8 (13) | 0 |
