# Supplementary material for: Chagas cardiomyopathy in Boston, Massachusetts: Identifying disease and improving management after community and hospital-based screening
Source: PLoS Negl Trop Dis. 2024 Jan 19;18(1):e0011913. doi: 10.1371/journal.pntd.0011913 (PMC10830043; doi:10.1371/journal.pntd.0011913)
Supplement: Attachment — Submitted filename: PLOS feedback_KR.docx [file pntd.0011913.s005.docx]

December 21, 2023

Re: Resubmission of manuscript *Chagas cardiomyopathy in Boston, Massachusetts: identifying disease and improving management after community and hospital-based screening,* D-23-01150

Dr. Shaden Kamhawi, Dr. Paul Brindley, and Dr. Peter Hotez

Editors-in-Chief

*PLOS Neglected Tropical Diseases*

Dear Editors,

Thank you for the opportunity to revise our manuscript, *Chagas cardiomyopathy in Boston, Massachusetts: identifying disease and improving management after community and hospital-based screening*. We would also like to express our thanks to the reviewers for the positive feedback and helpful comments for correction or modification. It is our belief that the manuscript is substantially improved after making the suggested edits.

Following this letter are the reviewer comments with our responses in italics, including how and where the text was modified. The revision has been developed in consultation with all coauthors, and each author has given approval to the final form of this revision.

Thank you for your consideration of this manuscript for publication in PLOS Neglected Tropical Diseases.

Sincerely,

Katherine Reifler, MD

Post-Doctoral Research Fellow

Boston University Chobanian and Avedisian School of Medicine

Section of Infectious Diseases

**Methods**

**Reviewer #1: This is a single centre retrospective cohort analysis of 109 patients with a diagnosis of Chagas disease at different stages. This is a descriptive observational study with no real hypothesis. The main objective was to describe the rate of cardiac complications reportedly related with Chagas disease in a cohort of Central American migrants living in a non-endemic region.**

*Thank you for this brief summary. We feel that there is a need to better characterize the cardiac complications of migrants with Chagas disease in non-endemic areas, especially for those from Central America given relatively little published evidence, in order to raise awareness of these health problems so that potentially life-saving treatments can be provided.*

**Reviewer #2: Classification: I'm not sure if the classification of asymptomatic equates with indeterminate Chagas, could the authors explain more? It seems that this was patient-reported although other possibilities (e.g. LVEF, ECG alterations) seem to be available. Had authors considered dividing by a different categorization, e.g. anyone with abnormal ECG findings or use AHA categories. Also, sometimes it was hard for the reader to orient which category was being described or analyzed at a given time.
Data analysis: The sample size seems large enough to support simple comparisons of proportions (chi squared), or Fisher's exact could be used for small cells. The value of this would depend a great deal on the categories above. For example, to see differences between indeterminate and patients with progression. (Or symptomatic vs. asymptomatic).**

*Thank you for pointing out this important clarification. For this analysis we chose to describe patients who were symptomatic (defined as those who reported symptoms potentially attributable to Chagas disease, as documented in the electronic medical record in infectious disease physician and/or cardiologist notes) versus asymptomatic (those who did not report any of the symptoms above) because we believe it is clinically relevant to understand the different cardiac manifestations in these two groups (line 170-172). For example, we found cardiac manifestations in several asymptomatic patients who may be underrecognized without asymptomatic screening. Unlike indeterminate versus determinate stages of Chagas disease, our definition of symptomatic versus asymptomatic does not require any ECG or echocardiogram interpretation. However, we also describe the ECG and echocardiogram characteristics in the symptomatic and asymptomatic groups. In this revision we added a description of the AHA stages of Chagas cardiomyopathy in each group (symptomatic and asymptomatic). This has been incorporated in the results section in* ***Table 4*** *(line 373).*

*We appreciate the reviewer’s comments on how it was sometimes difficult to follow which category was being described or analyzed. We removed Table 2, which added an additional category and described patients’ symptoms based on whether they were screened or tested for Chagas disease. To simplify the results in the revision we describe the frequencies of symptoms (line 205-210).*

*We appreciate the point of considering statistical proportions analyses. In this descriptive analysis p-value calculations were not performed due to the exploratory nature of the study, the small sample size, and the absence of specific hypotheses for formal hypothesis testing. The focus was on characterizing the study population. In addition, many of the diagnostics were performed on a subset of patients only, introducing bias to proportions analysis.*

--------------------
**Results
-Does the analysis presented match the analysis plan?
-Are the results clearly and completely presented?
-Are the figures (Tables, Images) of sufficient quality for clarity?

Reviewer #1: As this is a retrospective cohort study no pre-planned statistical analysis was performed. Findings are clearly reported, only descriptive as the sample is too small to perform any formal statistical analysis. Tables and figures are appropriate.

Reviewer #2: The results are comprehensive -- sometimes it was hard to oriented toward which category was being described.**

*Thank you for this helpful feedback, as described above we have tried to simplify the analysis to discuss differences between patients who were asymptomatic versus symptomatic throughout the manuscript.*

--------------------

**Conclusions
-Are the conclusions supported by the data presented?
-Are the limitations of analysis clearly described?
-Do the authors discuss how these data can be helpful to advance our understanding of the topic under study?
-Is public health relevance addressed?

Reviewer #1: The authors concluded that in this retrospective series of individuals with serologic evidence of t. cruzi infection of a population composed of mostly Central American migrants, the incidence of cardiac complications was high in a non-endemic region. The authors suggest that Chagas disease diagnosis
prompts cardiac evaluation which often identifies actionable cardiac disease and provides opportunities for prevention and treatment. The discussion is lengthy and addresses the need for a systematic assessment of cardiac function in patients with as serologic diagnosis of Chagas disease regardless of the presence of symptoms.**

**Reviewer #2: Yes, but the authors could comment more on treatment and care options for indeterminate patients.**

*While this analysis focused on the evaluation of cardiac involvement for people with Chagas disease, we agree that treatment of patients with chronic indeterminate infection is an important topic. In this revision we included the names of the two antiparasitic treatment options for patients in the indeterminate stage of Chagas disease in the introduction section (line 95-97) and clarified why it is important to identify patients in this stage for treatment (line 96-100).*

--------------------

**Editorial and Data Presentation Modifications?**
Use this section for editorial suggestions as well as relatively minor modifications of existing data that would enhance clarity. If the only modifications needed are minor and/or editorial, you may wish to recommend “Minor Revision” or “Accept”.

**Reviewer #1: Minor issues:**

**Line 199; In the patients with stroke under the age of 40 was the TTE normal?**

*In the patients with stroke under the age of 40, the TTEs were abnormal; both patients had left ventricular aneurysm identified on TTE. We have included these structural details in the revisions in the results section. (line 210-213)*

**Table 3. How many of the patients with Stroke had AF detected before and after the stroke? Were these all ischemic strokes? If so state this.**

*Three out of four of the patients had ischemic strokes due to embolism and one patient had a hemorrhagic stroke; this detail is now included in the manuscript “Stroke was diagnosed in 4 patients (4%), including embolic stroke in 3 patients and hemorrhagic stroke in 1 patient.” (line 209-210). Only one of the patients who had an ischemic stroke had atrial fibrillation identified before the stroke, while the other patients did not have atrial fibrillation identified before or after the stroke. These details are now included in the revision: “Notably, two of the patients who experienced strokes were women less than 40 years old without alternative risk factors for stroke other than left ventricular aneurysms identified on both patients’ echocardiograms and atrial fibrillation noted in one patient prior to her stroke” (line 210-213).*

**Line 238; Please provide further information of the ambulatory monitoring including duration of monitoring. Was the PVC burden calculated if so report this. When you indicate atrial arrhythmias what are you reporting? AF, Atrial flutter, atrial tachycardia? What do you mean by skipped beats, this is not a cardiology term, premature atrial complexes extrasystoles atrial or ventricular? Please clarify. Pause are defined how > 3s symptomatic, symptomatic > 6 s? Diurnal or nocturnal during sleep?**

*Thank you for pointing out these important clarifications. We have included additional information on the ambulatory monitoring in the manuscript, including the specific types of atrial arrhythmia, which are now broken down in* ***Table 3*** *(line 265). PVC burden ranged from “rare” in one patient to 8% of the monitoring time for another patient, which is now included in the manuscript: ”PVC burden ranging from rare to 8% of the monitoring time” (line 258-259).*

*The term “skipped beats” was the cardiologist’s description in the ambulatory monitoring report; after further review of the cardiologist’s notes these were determined to be sinus pauses and was recategorized in* ***Table 3*** *(line 260-262, 265).*

*16/24 patients had 30-day event monitors, 3/24 patients had 2-week monitors, and the remaining 5 patients had monitoring between 24 hours-3.5 days long. This section now reads “Monitoring was performed for 30 days in 16 patients (67%) and for 1-14 days in the remaining 8 patients (33%).” (line 255-256)*

*We have also included additional information in the manuscript about the patients with sinus pauses; it now reads, “One patient had sinus pauses throughout the day and night that were asymptomatic and between 3-7 seconds long; this information was unavailable for the other patient with sinus pauses.” (line 260-262).*

**Line 249 ETT; Did patients with chronotropic incompetence have any symptoms? Please report if this is the case. Similarly, patients with Exercise induced ischemia had any demonstrated obstructive CAD?**

*Of the 6 patients with chronotropic incompetence, 5 had symptoms (including palpitations, chest pain, and/or dyspnea) and 1 was asymptomatic. This information is now in the manuscript (line 277-278). Of the 5 patients with exercise-induced ischemia, 2 underwent left heart catheterization; one was found to have 3-vessel CAD and underwent CABG, while the other was found to have single-vessel CAD and underwent stent placement. The other 3 patients had stress tests that demonstrated partially reversible perfusion abnormalities. The details have been added (line 279-281).*

**Line 259 CMRI: Only 12 patients had CMRI, it is unclear what were the indications and why so many pf the symptomatic patients did not have this test, please elaborate. Similarly, please report the burden of LGE as you do mention the finding of LGE and fibrosis.**

*Thank you for this important comment which highlights the fact that there is no clear consensus on which patients should get a cardiac MRI (CMR) as CMR remains an expensive diagnostic modality. While our cardiologists often try to obtain them in many patients with Chagas disease, the reasons that symptomatic patients did not have CMR performed included: a) lost to follow up; b) still awaiting CMR (which can take several months to schedule at our institution currently); c) or the CMR was denied by their insurance.*

*A cardiologist reviewed all the CMR’s with LGE presence to comment on the burden of LGE on each CMR, which is now included in* ***Table 3*** *(and line 291-292).*

**Line 271: Cardiac Catheterisation: Only 10 symptomatic patients underwent catheterisation and only 30% had some obstructive CAD, no mention on treatment for these patients. Similarly, patients with no evidence of obstructive CAD "suggested" that Chagas was the cause. Please elaborate on how you came to this conclusion, did you evidence vasospasm, is this due to distal microcirculation disease? Any insights on the characteristics of coronary flow in these patients? i.e. slow flow ecstatic coronaries etc which have been reported previously in Chagas cardiomyopathy patients?**

*We appreciate these comments from the reviewer about further clarifications on these topics. Treatment of non-obstructive atherosclerotic CAD seen in 3 patients was not considered to be within the scope of this paper, which focuses more on treatment for Chagas-related cardiac pathology.*

*We agree with the reviewer’s comment that we can clarify why Chagas was “suggested” as the cause for cardiomyopathy in patients with evidence against CAD. This is now included in the manuscript: “Other typical nonischemic cardiomyopathy etiologies were ruled out for these patients (including thyroid dysfunction, iron studies, human immunodeficiency virus) and the diagnosis of nonischemic cardiomyopathy due to Chagas disease was a diagnosis of exclusion in the setting of positive serologies” (line 304-307). Data on the presence of vasospasm, distal microcirculation disease, or other characteristics of coronary flow were not available at the time of this analysis and in this retrospective cohort.*

**Line 282: 2 patients with ICD were admitted for ablation and cardioversion. I assume cardioversion was for AF as this is not treated by the ICD, ablation of VT? What other treatment strategies were tried prior to ablation? i.e. amiodarone or other antiarrhythmic therapy? As this is a merely descriptive report these details are essential.**

*Thank you for this feedback. We added the following additional information: “The first patient was intolerant to three anti-arrhythmic medications (amiodarone, sotalol, and dofetilide), developed refractory atrial fibrillation despite pulmonary vein isolation that was revised twice, and was admitted several times for cardioversion. The second patient was admitted for ablation after receiving two asymptomatic shocks at home for rapidly conducting supraventricular tachycardia” (line 317-321).*

**Discussion**
**Line 346 ref 23 is a bit outdated several more recent studies have reported the Global Longitudinal strain detected by TTE has a significant predictive value for detecting early cardiomyopathy in patients with Chagas initially classified in Stage A.**

**Echeverría LE, Rojas LZ, Villamizar MC, et al Echocardiographic parameters, speckle tracking, and brain natriuretic peptide levels as indicators of progression of indeterminate stage to Chagas cardiomyopathy. Echocardiography. 2020 Mar;37(3):429-438. doi: 10.1111/echo.14603. Please review and update references.**

*Thank you for highlighting this study about the technique of speckle-tracking echocardiography to obtain left ventricle global longitudinal strain, which some studies have shown to better predict future development of Chagas cardiomyopathy. We do not routinely do LV strain on the echocardiography for our patients with Chagas disease. In addition, raw image acquisition was not available to retrospectively apply strain software algorithms, so LV strain could not be performed uniformly in this dataset. We will look to applying LV strain for future studies. We have included discussion of LV strain in the discussion section of the revision (Line 394-396).*

**Similarly, the BENEFIT trial published a Echo subsidy that identified a simple and reproducible Wall Motion's Score that predicted mortality and cardiovascular events. Please review and include this reference.**

**Schmidt A, Dias Romano MM, Marin-Neto JA, et al. Effects of Trypanocidal Treatment on Echocardiographic Parameters in Chagas Cardiomyopathy and Prognostic Value of Wall Motion Score Index: A BENEFIT Trial Echocardiographic Substudy. J Am Soc Echocardiogr. 2019 Feb;32(2):286-295.e3. doi: 10.1016/j.echo.2018.09.006.**

*We agree that the wall motion score is another potentially promising echocardiographic calculation to predict poor outcomes due to Chagas cardiac disease. We have now included these references in our discussion: “Echocardiography techniques such as calculation of left ventricle strain or the BENEFIT trial’s wall motion score may better predict development of Chagas cardiomyopathy or cardiovascular events, respectively, but these are not routinely performed.” (line 394-396). In addition, we mentioned the inability to report LV strain and wall motion’s score as limitations of the analysis (line 456-457).*

**Reviewer #2:**

**Abstract: In Results, it would be good to have absolute numbers for the numerators and denominators along with the percentages. (For example do the percentages of symptoms reflect the entire sample or just the 21% who were diagnosed due to symptoms and ECG abnormalities?)**

*Thank you for this suggestion. In this revision we added numerators and denominators in the results section of the abstract for clarity (lines 43-53).*

**63-64: Maybe this statement should be contextualized more, since there are PAHO and AHA recommendations and consensus guidelines in Brazil and elsewhere. Guidelines for the Diagnosis and Treatment of Chagas Diseases (paho.org), 2nd Brazilian Consensus on Chagas Disease, 2015 - PubMed (nih.gov), Chagas Cardiomyopathy: An Update of Current Clinical Knowledge and Management: A Scientific Statement From the American Heart Association | Circulation (ahajournals.org)**

*Thank you for this feedback; it is true that the guidelines recommend two serologies for Chagas, history, physical exam, an ECG, and a 30-second rhythm strip, and “additional cardiac…workup is recommended only when this first evaluation raises clinical suspicion of more advanced disease forms.” We updated this sentence to say, “The 2018 American Heart Association guidelines recommend ECG and cardiac rhythm strip, but there is no consensus on the approach to further cardiac evaluation in the US, or management of Chagas cardiomyopathy.” (line 64-67).*

**80-81: We don’t really have evidence that it’s growing in the 2020’s (and reference 3 wouldn’t really support that statement), although we do have an estimate of the burden in people of Latin American origin from the US as mentioned already – I would just leave it at that, and mention that the US also has limited, occasional evidence of both congenital and autochthonous vector transmission.**

*We agree that it is difficult to estimate whether the burden of Chagas disease is growing in the US due to several factors including underdiagnosis and migration of undocumented people who may not be counted. We have edited the sentence to say that “the number of affected individuals in the US and other countries may increase with current migration trends” and we also added your important point that other forms of infection in the US include congenital and autochthonous vector transmission (line 82-84).*

**93-95 and is also not recommended in current PAHO recommendations**

*This comment is referring to our description that “in patients with established cardiomyopathy, antiparasitic treatment does not seem to prevent cardiomyopathy progression”. We agree with the reviewer’s comment that treatment is not recommended, which corresponds with our statement.*

**126 – could include what test was used at CDC (Wiener rec. v. 3?, and any other test to confirm positives?)**

*This revision now includes additional details and citations in the methods section for the specific types of testing performed at the CDC (the Wiener rec. v.3. and/or laboratory developed TESA-blot): “Patients received an initial positive Chagas screening ELISA test performed by a commercial laboratory and a confirmatory test at the Center for Disease Control [the Wiener Chagatest recombinante v.3.0 enzyme immunoassay (EIA; Wiener Laboratories, Rosario, Argentina) or the trypomastigote excreted-secreted antigens (TESA)-blot, with discordant results mediated by an immunofluorescence assay” (line 130-137).*

**157 “was” classified**

*Thank you for catching this error, this has been changed to “was classified” (line 162).*

**156-159: definition of symptomatic was whether symptoms suggestive of Chagas disease were reported by the patient? Or this was based on review of patients’ medical records? For Table 1, I wonder if you considered dividing by a different categorization, e.g. anyone with abnormal ECG findings or use AHA categories.**

*Thank you for this comment. I added a clause in the study definitions to clarify that the definition of symptomatic was based on review of patients’ medical records after infectious disease and cardiology evaluation (line 159-160).*

*For* ***Table 1****, as explained above, for this analysis we chose to describe patients who were symptomatic versus asymptomatic (without incorporating any ECG findings) because we believe it is clinically relevant to understand the different cardiac manifestations in these two groups to underscore the importance of asymptomatic screening. For example, we found cardiac manifestations in several asymptomatic patients who may be underrecognized without asymptomatic screening. We also reported the ECG and echocardiogram characteristics of the symptomatic and asymptomatic patient. In this revision, we report the AHA categories of the symptomatic and asymptomatic groups, which is used for determining treatment eligibility (****Table 4****, line 373).*

**Figure 1: the text and caption indicate 25, not 52? Some patients have multiple findings?**

*This may have been written in a confusing way previously; the text and caption indicate that 52 intervenable cardiac findings were found in 25 unique patients, meaning that some patients had multiple potentially intervenable findings. The manuscript text and* ***Figure 1*** *caption (line 346) have been updated to minimize confusion. The manuscript now says “Fifty-two intervenable findings were found in 25 (23%) unique patients, with some individuals having multiple abnormalities” (line 325-326).*

**345-347: Interesting finding, how does it compare to what is in the literature?**

*This comment refers to our finding that “Echocardiographic abnormalities were seen in 31% of patients with normal ECGs and led to change in management in 16% of patients, suggesting that echocardiography should be routine and, ideally, include contrast” (now line 387-389). Echocardiogram is not routinely recommended in all patients who have normal ECGs, and therefore most of the literature available reports echo findings in patients with more severe forms of disease (such as cardiomyopathy), whereas less is known about echocardiogram findings in patients with normal ECGs. However, the 2018 AHA guidelines state that “global radial strain [assessed by echocardiogram] may be decreased in some patients in the indeterminate stage, even with a normal ECG,” which supports our findings. In addition, a recent Spanish study reported that 16/148 (10.8%) of patients with Chagas cardiomyopathy had normal ECGs and were diagnosed with cardiomyopathy by TTE (Citation: Laynez-Roldan P, Losada-Galvan I, Posada E, de la Torre Avila L, Casellas A, Sanz S et al. Characterization of Latin American migrants at risk for Trypanosoma cruzi infection in a non-endemic setting: insights into initial evaluation of cardiac and digestive involvement. PLoS Negl Trop Dis. 2023;17(7): e0011330).*

*In the revisions we included a summary of some of these results in the discussion (line 391-393).*

**386: does this 51% reflect the “asymptomatic” group from Table 1?**

*The statement that “over half (51%) of our patients were in the indeterminate stage A of disease” (now line 434) refers to the findings in* ***Table 4*** *(previously Table 5); in this statement we are referring to the standard AHA definition of stage A indeterminate disease (not the asymptomatic group) because these stages are used to determine whether a patient may benefit from anti-trypanosomal therapy.*

**Discussion: maybe you could also comment more on why it is important to identify patients in the indeterminate stage, and what options for treatment are available.**

*Thank you for pointing out this important clarification. In the introduction we explained that “early antitrypanosomal treatment may prevent Chagas cardiomyopathy development for patients in the indeterminate stage” (line 96-97). In this revision we specify that “early” means for those in the indeterminate stage of disease, and we have specifically named the antiparasitic treatment options, benznidazole and nifurtimox (line 95-96). The introduction further explained the importance of identifying patients in the indeterminate stage: “in patients with established cardiomyopathy, current data suggest that antiparasitic treatment does not seem to prevent cardiomyopathy progression and cardiac treatment options are focused on standard heart failure therapies” (line 97-100). This is reiterated in the discussion by saying, “for…patients in more advanced stages of cardiomyopathy, antitrypanosomal therapy does not seem to prevent death or major cardiovascular events,” (line 438-440) with citation of the BENEFIT trial.*

--------------------

**Summary and General Comments**
Use this section to provide overall comments, discuss strengths/weaknesses of the study, novelty, significance, general execution and scholarship. You may also include additional comments for the author, including concerns about dual publication, research ethics, or publication ethics. If requesting major revision, please articulate the new experiments that are needed.

**Reviewer #1**: **This is a retrospective cohort study that included 109 patients with Chagas disease at different stages. Strengths include that for the US this is a large sample and highlights the fact that almost half of the patients ended with some form of cardiomyopathies alteration. This is worth highlighting. On the other hand the study has several limitations with many patients having very few diagnostic tests which limits the use of this data to provide any sort of recommendation on which should be the appropriate diagnostic approach on this often selected population. The authors should perform a more systematic approach and propose an approachable diagnostic algorithm based on their findings. Both the role of GLS and calculating the Wall Motion Index score suggested above should be performed and the manuscript adjusted accordingly.**

*We agree that a limitation of this retrospective chart review is that cardiac diagnostics were not prospectively obtained nor were they obtained in a systematic and uniform way. Additional metrics such as global longitudinal strain and wall motion score were not able to be added due to the following reasons: a) these were clinically obtained echocardiograms and these metrics were not obtained prospectively; b) due to technical limitations and lack of raw images we are unable to retrospectively obtain global longitudinal strain with our current vendor strain package; and c) there is some variability in the quality of the endocardial resolution to accurately go back retrospectively to calculate a wall motion score and are additionally limited by the time frame requested by the reviewers for this single metric.*

*We agree with the Reviewer 1 that a diagnostic algorithm could be considered with the additional echocardiographic metrics, however, this is beyond the scope and purpose of this current manuscript. We do believe our findings support thorough cardiac evaluation for patients with Chagas disease, regardless of symptoms, including echocardiogram with contrast in every patient and strong consideration of cardiac ambulatory monitoring, which we emphasize strongly in the discussion (line 384-389, 408-410).*

**Reviewer #2:** **This is an interesting, well written and important study following a cohort of patients with Chagas disease in Boston. The study makes an important contribution to the literature on Chagas disease in the US. The different classifications used were sometimes hard to follow; the paper could be strengthened by choosing one classification based on the literature (e.g. AHA stages?) and just ensuring the structure of the paper facilitates clarity about which group is being described where.**

*Thank you for this helpful comment which encouraged us to simplify the classifications that we use throughout the paper. We decided to focus on one classification based on the presence of symptoms versus asymptomatic patients because we believe this approach best highlights the implications of asymptomatic screening. We believe the classification of screened versus tested may have confused readers and so we limited use of this classification in the manuscript revision. We used the AHA classification only when describing which of our patients may benefit from antiparasitic therapy because this is the clinical standard to determine treatment approach in the literature.*
